# Mentoring and Supporting Our Next Generation of Women Toxicologists

**DOI:** 10.3389/ftox.2022.920664

**Published:** 2022-06-30

**Authors:** Hollie I. Swanson

**Affiliations:** Department of Pharmacology and Nutritional Sciences, University of Kentucky, Lexington, KY, United States

**Keywords:** women, toxicologists, career, advancement, mentoring

## Abstract

Career success of women toxicologists requires intentional strategies designed to encourage and support their professional and personal growth. Key among these are mentoring approaches which should be initiated early in their academic careers and continue as their careers progress. While undergraduate and graduate students as well as postdoctoral fellows, women engaged in all STEM fields benefit from one-on-one mentoring experiences offered by both their peers, near-peers and faculty. Here, they not only receive encouragement and lessons on “how to be a good mentee”, but also gain scientific and life skills. Networking opportunities and career planning advice are also important benefits. As woman scientists progress in their careers, they continue to benefit from one-on-one mentoring and structured career development programs adapted to meet their changing needs ultimately culminating in leadership coaching as they reach the pinnacles of their careers. While mentoring success is best facilitated by structured programs that match mentees with mentors and offer training, support and programming, the availability of these programs to women toxicologists is limited. Opportunities for women to participate in structured mentoring programs should be enhanced by institutions, funding agencies and scientific societies as a component of accelerated diversity and inclusion efforts.

## Introduction

The success of the next generation of women toxicologists requires an assessment of their future needs and challenges to allow for a correspondingly restructuring of the scientific community to maximize their training and support. The impetus for change arises from the fact that our academic and work environments were founded by men and traditionally best accommodate their needs and work styles (Vasic 2021). As a consequence, women often report feelings of isolation and marginalization at all stages of their careers and cite difficulties in balancing societal expectations versus career needs as well as a lack of appropriate role models. Ultimately, they may become less engaged and are at greater risk of experiencing career burn-out. Individuals who have been historically underrepresented in the scientific community, as well as those who are first generation college students often report similar experiences. Unfortunately, these commonly experienced barriers often persist along the continuum of a woman’s career (O’Connell and McKinnon 2021). A key and effective approach that has been shown to enhance the participation of women and those from other underrepresented groups in the scientific community is quality mentorship (Haeger and Fresquez, 2016; Hernandez et al., 2017; Estrada et al., 2018). In this way, a more inclusive community of scholars who share a common science identity can be created.

Mentoring harbors both relationship and developmental aspects that typically address both career and psychosocial needs (Mullen and Klimaitis 2021). Traditional mentoring wherein a junior protégée is dyadically paired with a more senior advisor typically focuses on career progression and is facilitated by one-way communication. More recently, mentorship relationships have been expanded to encompass multiple structures in large part to address problems that may arise due to power hierarchies and constraints pertaining to diversity and cultural differences (National Academies of Sciences and Medicine, 2019). These mentoring structures may take on a variety of configurations including triads, collective or group mentoring and mentoring networks. The involved mentors may be peer or near-peer colleagues as well as multiple senior colleagues who can work collaboratively to enhance the breadth and depth of career and psychosocial mentoring functions. While in person, face-to-face interactions are often preferred, virtual environments can provide unique advantages including enhanced access to alumni and to a range of scientific experts (Tinoco-Giraldo et al., 2020). By engaging in a rich variety of mentoring configurations formed via informal or formal means, a woman toxicologist can better meet her ever changing range of needs and receive the necessary advice and support for successfully navigating both her career and life challenges.

## Nurturing the Mentoring Relationship

Mentoring often occurs within formal/organized or informal/ad hoc structures (National Academies of Sciences and Medicine, 2019). Traditionally, mentoring relationships have formed via informal mechanisms, often at the initiative of the mentee and with a more senior colleague. Formal mentoring, occurring within specifically designed, structured programs is becoming more frequently used as a mechanism to promote inclusivity and to promote quality mentoring (Guerrero et al., 2017). Requirements for quality mentoring include identifying effective mentors, facilitating an appropriate match between the mentor and mentee, providing training on how to be good mentors/mentees, establishing guidelines for clear expectations of roles, commitment to the mentoring relationship, and a supportive structure to facilitate networking and other opportunities (Pfund et al., 2016). In addition, mentors engaged in negative mentoring, which includes failure to honor time commitments or undermining the mentoring bond must be identified relatively early within the mentoring relationship and their role within the mentoring program must be reconsidered. Given that quality mentoring often requires a significant time commitment, that women are increasingly asked to perform mentoring roles and the high risk of burnout faced by women in the workplace, it is essential that mentoring transition from “invisible, but expected” work and incorporated into formal job responsibilities with clear expectations (Burns et al., 2021). A key aspect of successful mentoring is a proactive mentee who seeks out specific mentors, sets and manages their mentoring expectations, approaches the mentoring relationships with a positive attitude and intention and takes charge of their own career development (Sarabipour et al., 2022).

What are aspects of a quality mentoring relationship/a good mentoring match? Efforts to understand the factors that contribute to quality mentoring have often focused on how well the mentor and mentee “mesh” with respect to their personalities, perspectives and expectations. It is important to understand the mentoring relationship as a collaboration wherein both the mentees and mentors engage as “active learners” (Pfund et al., 2016). Personalities traits often described as the “Big Five” (emotional stability, extraversion, openness to experience, agreeableness and conscientiousness) of both the mentee and mentors play important roles. With respect to the ability of a mentee to obtain and receive quality mentoring, personality, emotional stability was found to be amongst the most consistent predictors (Bozionelos et al., 2014). Mentees who perceive that their values are similar to those of their mentors are also most likely to report mentoring success (Illies and Reiter-Palmon 2018). With respect to characteristics of “good mentors”, a survey of graduate students indicate that the most desired qualities in a mentor are communication skills and providing feedback whereas top mentoring attributes are integrity, guidance and relationship (Rose 2003). This latter study informed the development of the “Ideal Mentor Scale” which may be used to assess mentoring quality (Sozio et al., 2017). It should also be noted that for women mentees, having women mentors as role models and being able to engage in a same-gender mentorship relationship has been highly successful in advancing the careers of women scientists (Deanna et al., 2020).

## Beyond Mentoring

In addition to mentoring, other forms of support that can play key roles in advancing women toxicologists are coaching and sponsorship. Coaching pertains to support in learning a specific skill and typically involving practical work performed over a short period of time followed by feedback that specifically addresses the performance of the individual (Clutterbuck 2008). While the definitions of coaching, mentoring and sponsorship are often intertwined, coaching may be viewed as “helping”, mentoring as “giving” and sponsorship as “investing” (Ang 2018). An example of coaching used to address the career needs of scientists is a group coaching intervention designed to enhance success in grant submissions (Weber-Main et al., 2022). Coaching is also being used to enhance educational efforts of faculty and at the executive level, for leadership development (Kirk et al., 2019; Jordan et al., 2021). Current thinking regards sponsorship (or sometimes “championship”) as even more important than mentorship given the heightened focus of sponsors on professional development behaviors and contributions to three key competencies to career success “know–why”, “know-how” and “know-whom” (Ang 2018). Similar to that of a sponsor, a “champion” is highly committed to the success of their protégé, is well connected and willing to challenge the status quo (Gallop and Chamorro-Premuzic 2021). While sponsorship has been shown to play a key role, especially for women, in achieving career advancement, the sponsorship relationship involving women appears to differ from that of men (Levine et al., 2021). Here, women are less likely to seek out sponsorship and less likely to receive sponsorship. Addressing these types of issues to advance the careers of women toxicologists will likely require more structured programs that can incorporate “best practices” and formalize these types of relationships.

## Adapting Mentoring and Support as Careers Progress

As women progress in their careers, their needs, expectations and benefits with regards to mentorship will undergo corresponding changes. Quality mentoring relationships that appropriately adapt to these changes will benefit not only the mentee, but also the mentor and the scientific community. Mentors receive a number of benefits from the mentoring relationship that include increased productivity, increase in engagement in the workplace and scientific community and a heightened of belonging and professional value. The scientific community benefits from the increased scholarship, creativity and vibrancy. The following sections will focus primarily on mentoring during different stages of women’s academic careers, but many of these concepts can be generalized to other sectors of the workforce.

### Mentoring Undergraduate Students

It is well established that undergraduate students who are involved in a mentoring relationship experience better retention in their programs of study, are more satisfied with their choice of academic program and more likely to attain measures of success (i.e., higher exam scores) (Jacobi 1991; Campbell and Campbell 1997). Undergraduate students report a need for mentors who provide 1) support and encouragement thereby creating an emotional safety net and 2) constructive feedback as the student explores career options and sets goals (Law et al., 2020). It has also been suggested that a great mentor to undergraduates is one who can show them the big picture, introduce them to the literature, offer ownership and provide them with a stage to speak about their work (Deshpande 2017). Student participation in undergraduate research experiences provide major opportunities for engaging in mentoring relationships within STEM programs and when coupled with intentional mentoring that provides socioemotional support as well as skills-based training, can be highly effective for encouraging students from diverse population groups to participate in scientific endeavors (Haeger and Fresquez; Byars-Winston et al., 2015). For women undergraduates, a key benefit of mentoring-development of a mentee’s scientific identity, can be particularly enhanced when they have access to women mentors (Hernandez et al., 2017). Interestingly, women versus men mentees place a higher preference on the relational aspects of mentoring (Rose 2005). However, it is important to note that undergraduates struggle in knowing how to find a mentor and those who place a high value on mentoring appear to be more discerning in establishing positive mentoring relationship (Wright et al., 2022). This type of “hidden curriculum” disproportionally affects first generation and under-represented groups, but can be addressed by incorporating soft skills such as “how to be a good mentee” into the STEM coursework (Wrighting et al., 2021). For women toxicologists, the undergraduate years present a critical window of opportunity wherein the most savy, well-informed and well-advised individuals are able to participate in mentoring relationships to form a strong foundation for building their future careers.

### Mentoring Graduate Students

Mentorship during an individual’s graduate career is positively linked to a mentees scientific impact (Ma et al., 2020). Given that a scientist typically engages in the highest level of contact with mentors during their graduate training, these mentors are often key gatekeepers to a mentee’s scientific success. It has been previously proposed that faculty mentors play three major roles as *allies*, *ambassadors* and *master-teachers* (Lechuga 2011). As *allies*, mentors focus on the individual needs (academic or otherwise) of their mentees and take a supportive approach towards their working relationship. As *ambassadors*, faculty introduce their students to their scientific discipline, instill a sense of professional identity and familiarize their students with the types of activities their future careers will entail. As *master-teachers*, faculty allow students to work relatively independently to demonstrate their research abilities and become expert researchers. Early-career mentees are advised to engage with multiple mentors who are able to provide perspectives from a variety of backgrounds and experiences as well as expertise in specific skills (Sarabipour et al., 2022).

A number of issues that currently impact graduate students raise concerns regarding the future of women toxicologists and highlight the need for significant reenvisioning of our structures and support of their budding careers. Despite the importance and value of mentoring, some, in particular traditionally underrepresented women report a lack of availabilities of mentors which can significantly impact our efforts to enhance diversity and inclusion (Guy and Boards 2019). Some reports indicate success in using peer mentoring programs to address these issues (Levy-Tzedek et al., 2018). An additional concern is that interest amongst graduate students to pursue faculty positions is in decline with women from traditionally underrepresented populations demonstrating the least interest and gender bias within the academic environment persisting (Gibbs et al., 2014; Remich et al., 2016; Wood et al., 2016). Many graduate students, in particular those from traditionally underrepresented populations harbor significant concerns regarding the extent to which an academic career impacts on work/life balance. Incorporating opportunities for career development via experiential learning (i.e., job simulations, employer site visits, shadowing and internships) can allow for more intentional career-planning discussions (Van Wart et al., 2020). Success in these types of endeavors for women toxicologists would be greatly enhanced by incorporating women mentors from all sectors of the workplace into these types of academic programs.

Mental well-being of graduate students is a key concern with many students reporting emotional exhaustion and depression as they conduct their research projects (Rigg et al., 2013; Gin et al., 2021). Some of these issues can be addressed by improving and supporting mentoring such that mentors can better normalize struggle and failure to promote a growth mindset (Posselt 2018). Additional measures that mentors can undertake include breaking down research projects into smaller tasks, increasing collaborative work, offering work flexibility, and ensuring that the work aligns with a mentee’s interest or passions. Mentors can also ensure that their mentees are making good progress and receive appropriate emotional support. Finally, our academic institutions must be more effective in addressing bullying and harassment to create a more positive research culture that exemplifies diversity, collaboration, transparency, value and support of the contributions of individual researchers and nurture creativity (Lancet 2022).

### Mentoring Early Career Faculty

Early-career STEM faculty face many challenges that arise from the increased emphasis on research success, the highly competitive research environment, enhanced scrutiny on “student success” and an often overwhelming workload (Hollywood et al., 2020). Early career STEM women faculty also cite a “chilly” working environment and experiences of ostracism and incivility from their male colleagues and are susceptible to higher workloads due to expectations of more service and other “academic housekeeping” activities (Macfarlane and Burg 2019; Miner et al., 2019; Casad et al., 2021). These challenges are due in part, to the fact that STEM women faculty are still underrepresented within their disciplines and departments. Efforts to address these issues include strategies to increase success in recruiting from a diverse application pool (i.e., changes in recruiting efforts and training search committee members on best practices), institutional mentoring and networking opportunities and interventions to improve the academic climate. Effective mentoring has been shown to be very beneficial in advancing female academic careers whereas inadequate mentoring often leads to isolation, limited career development, job dissatisfaction, burn-out and attrition (Cross et al., 2019). Institutional efforts to address these issues include programing to provide structured mentorship, sponsorship, and networking opportunities involving newly recruited and established STEM women faculty and administrators (Jamison-McClung 2022).

To achieve success in the tenure track at research-intensive institutions, it has been recommended that early-career STEM faculty should engage in long-term strategic planning as early as possible (Boyce and Aguilera 2021). However, life events which may require extended periods of leave (e.g., family leave) may contribute to uncertainties and limit the effectiveness of such planning. Measures in addition to mentoring, that can be undertaken by institutional leaders include allowing early-career faculty to focus on developing their research programs by minimizing teaching expectations and clearly defining expectations for teaching, service and research activities (Sawarkar et al., 2019). Research support should include sufficient start-up funds and equipment, availability of senior faculty to provide constructive feedback and assistance in developing grant proposals. Finally, to develop a sense of community and sense of belonging, leaders should cultivate a supportive and collegial environment. Early-career STEM women faculty can also enhance their productivity and sense of community by participating in faculty writing groups that typically involve structured writing sessions and peer feedback (Kwan et al., 2021). Bringing awareness to the specific challenges facing early career women toxicologists and addressing them with specific interventions is key for ensuring the vibrancy of our toxicology community.

### Mentoring Mid-Career Faculty

In general, mid-career professional women face three major career issues; authenticity, work/life balance, burn-out and lack of challenge-driven opportunities for professional growth (Burns et al., 2021). As a consequence, a women’s career may stagnate thereby adversely impacting institutional efforts in achieving equity and diversity. The mid-career stage is the window of opportunity wherein the leadership “track” typically begins, yet many individuals and in particular, women, are poorly prepared or lack aspirations for entry point leadership positions (Templeton and O'Meara 2018; Baker et al., 2019). Many organizations have attempted to address these issues by supporting women-only leadership development programs (Hopkins et al., 2008; Clarke 2011; DeFrank-Cole et al., 2016). In these programs where women are in a majority position, a “safe” environment can be created wherein participants can openly share their frustrations and challenges. The development of women leaders should include assessment of leadership competencies, training and education with respect to leadership skills, coaching and mentoring, networking, career planning and experiential learning. However, these programs may be limited in effectiveness if they fail to address the realities that women face in their own organizations and fail to provide growth opportunities for aspiring women leaders. It is important to recognize that the leadership development needs for women are unique (Gipson et al., 2017). As compared to men, women exhibit differences in behaviors and leadership styles. For example, women leaders are often credited with more democratic and transformational leadership styles. In addition, performance evaluation of women leaders are often more scrutinized. Thus, the most effective programs can bring awareness to the unique leadership developments needs of aspiring women leaders and develop strategies for advancing their career progression. When closely aligned and integrated with the strategic objectives of the organization, they can create much needed opportunities for organizational transformation (Debebe 2009).

**FIGURE 1:**
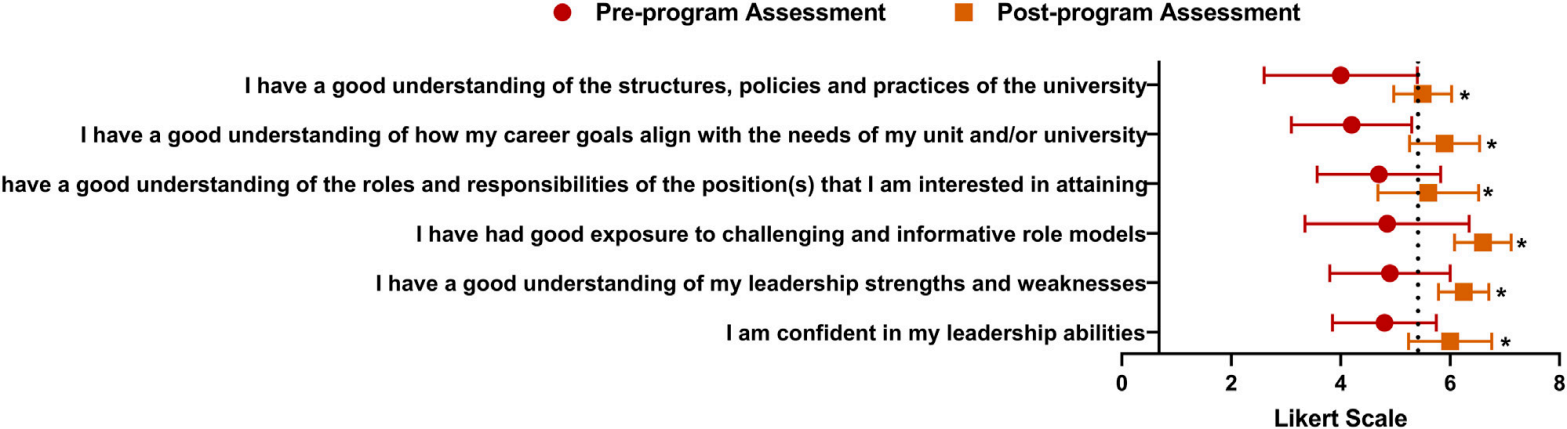
A snaphot of self-reported gains in measures of confidence, leadership, career aspirations and institutional knowledge following participation in a women’s leadership development program. Electronic surveys were administered to the 2019 cohort (n = 25) of the Women’s Executive Leadership Development Program held at the University of Kentucky prior to and following participation in the 8 month program. Respondents rated the degree to which they agreed or disagreed with the indicated statements using Likert-scale categories from “strongly disagree” through “strongly agree”. The Likert scales were adjusted to numerical scores of 1 (“strongly disagree”) through 7 (“strongly agree”), the mean Likert scores and standard deviations were calculated for each question and statistical differences were identified by utilizing an independent samples *t*-test. The dashed line indicates a neutral (“neither agree nor disagree”) response. Pre-assessment responses = 20 and post-assessment responses = 8. *Significantly different (*p* < 0.05).

The Women’s Executive Leadership Development program that I established at the University of Kentucky was designed to address barriers that mid-career women faculty and staff may face in attaining leadership positions. Prior to participating in the program, individuals representing a variety of backgrounds and disciplines, self-identified their needs which typically involved building their network, confidence, focus and value as well as specific skills such as leading change, managing others, negotiating and managing budgets. Testimonials from program participants indicated that sessions most valued were those that addressed conflict management, negotiations and budget management that the most beneficial aspects were networking opportunities and meeting women who are in a variety of roles across the campus. These networks often formed the basis for establishing mentoring relationships as well as sponsorship. An example of gains obtained by program participants that were revealed in our pre- and post-assessment surveys is shown in Figure 1. Here, a single cohort of program participants self-reported gains in measures of confidence, specific leadership skills and institutional knowledge which are key for effectively navigating the organizational culture. These findings are consistent with those reported from similar women—only leadership development programs (Hopkins et al., 2008; Clarke 2011; DeFrank-Cole et al., 2016). Similar programs could be within scientific communities and other organizations to develop leadership of women toxicologists.

### Mentoring and Seasoned/Faculty/Senior STEM Women Leaders

Senior faculty retain positive views regarding mentoring as it provides them with a means of leaving a legacy and maintaining a professional identity (Grosshans et al., 2003). By engaging in mentoring relationships, senior faculty can maintain career vitality, as indicated by a recent survey. Here, four critical strategies were cited; 1) engaging in sustained relationships with students and others, 2) practicing self-efficacy to allow them to manage their workload and prioritize their work, 3) maintaining a positive/growth mindset and 4) engaging in the scholarly life (Cruz and Herzog 2018). Senior faculty can also participate in reverse mentoring. Reverse mentoring is increasingly being used to increase retention of the younger generation (i.e., Millennials) as well as enhance digital skills of the more senior mentees (Morris 2017; Jordan and Sorell 2019). Other potential advantages of reverse mentoring include a positive impact on driving cultural change within the organization, promoting diversity and enhancing teaching effectiveness. Senior STEM women leaders report numerous barriers to leadership, including balancing work/home life, imposter syndrome and devaluing of achievements (McCullough 2020). They are often prone to working in isolation and most commonly rely on spouses/partners as well as peers for support and encouragement. Strategies used to address these issues include engaging in peer mentoring to form a “mutual mentoring” program (List and Sorcinelli 2018). Thus, even at an advanced career stage, women toxicologists can benefit from mentoring by serving as a mentor, participating in reverse mentoring and engaging in peer or mutual mentoring.

## Do Women Toxicologists Face Unique Challenges?

The future of women toxicologists is strong as indicated by the relatively high interest of women in obtaining doctoral degrees in toxicology (Gillen and Tanenbaum 2014). Interestingly, toxicology (as well as pharmacology) are the most gender-balanced amongst the biology and biomedical fields with respect to doctoral degrees awarded. Women’s interest in toxicology remains high as they progress towards postdoctoral fellowships with women representing 42% of those who study within pharmacology/toxicology areas and pharmacology/toxicology represented at levels comparable to those in similar disciplines such as biochemistry or physiology (https://ncsesdata.nsf.gov/home). Thus, our expectations that the status of women toxicologists will continue to improve remain high.

While our understanding of mentoring and how it may be best used to promote the development and growth of individuals at a range of career stages and from diverse backgrounds is growing, the mentoring literature is still dominated by four disciplines; academic medicine, industrial and organizational psychology, education, nursing and psychology (Lefebvre et al., 2020). Effective mentoring must address the specific barriers to career progression that may be encountered within each discipline. A brief review of the literature suggests that many of the challenges faced by women toxicologists are similar to those reported within other scientific fields and disciplines. For example, women in neuroscience report challenges associated with subtle biases and stereotypes including frequent interruptions during talks and seminars, underrepresentation as authors in high-profile journals and gender gaps in salary (Machlovi et al., 2021). Similar issues have been noted by women in physiology (Gordon 2014), cell biology (Gieniec 2022), zoology (Slobodian et al., 2021) and other STEM disciplines (Birnir and Eliasson 2018; Rosser 2018). Unfortunately, the COVID-19 pandemic has exacerbated many of these challenges and contributed to greater gender inequities within the academic environment (Malisch et al., 2020). The gender pay gap has remained consistent, however, with women overall earning 86% of what men earned in 2021 (Fry 2022). Like women in other scientific disciplines, women toxicologists are compensated at lower levels than their male counterparts within all employment sectors (Sullivan and Gad 2020). Addressing challenges faced by women toxicologists requires heightened visibility and consistent reporting as exemplified by the “Perspectives of Women in Toxicology” session held at the ICTXV2019 meeting and reports by groups such as the Women in Toxicology of the gender gap for awards within the Society of Toxicology (Lewis 2019). Similar successful efforts to address issues faced by women toxicologists and facilitate systemic change and gender equality are being undertaken by Women in Toxicology within the American College of Medical Toxicology (Spyres et al., 2019).

A few aspects that contribute to unique challenges experienced by women toxicologists should receive greater scrutiny by our professional societies. Early in their academic careers, women toxicologists may face exceptional difficulties in identifying women mentors due to the absence of representation of toxicology within the curriculum of undergraduate-serving communities. In addition, because of the highly interdisciplinary nature of toxicology which spans the full gamut of scientific fields ranging from Earth sciences, biomedical sciences and engineering to social and clinical sciences, potential women mentors are often housed in a variety of academic units making them difficult for a young woman STEM major to locate. Finally, because of the underrepresentation of women at the senior level of faculty ranks within biomedical disciplines of research-intensive universities (Hamrick 2019) the pool of women mentors available to these potential early career women toxicologists is quite small. These issues may also affect women toxicologists as they progress in their academic careers. Here, they may become significantly isolated as they experience difficulties in identifying mentors and supporters who understand their unique needs, opportunities and challenges. As a result, career dissatisfaction may arise as more mentoring of women scientists by other women scientists corresponds to a greater sense of “voice” or ability to influence their work environment as well as a reduced perception of negative work environments, increased connectedness and heightened sense of community (Ragins 1997; Settles et al., 2007). Women toxicologists may also face difficulties in obtaining funding and publishing their work if they study topics that are “gender-specific” and affect primarily women (Mirin 2021). As we further evaluate these unique challenges, we can then develop more specific measures to ameliorate their impact.

## Conclusion

While women toxicologists have made significant strides with respect to improving the quality and advancement of their careers, significant challenges persist. Mentoring can, in part, address some of these challenges. However, to be effective for enhancing the status of women toxicologists, the needs and potential benefits must be adapted to each woman’s career stage. In many cases, mentoring should be coupled with additional interventions that include coaching and sponsorship and must be valued within the workplace. Scientific communities, funding agencies and academic institutions must work coordinately to provide mentoring opportunities and structures that promote the career advancement of women toxicologists.

## Data Availability

The original contributions presented in the study are included in the article/supplementary material, further inquiries can be directed to the corresponding author.
